# The Father's Responsibilities

**Published:** 1920-08-07

**Authors:** 


### The Father's Responsibilities.

The responsibility of the father was considered in several relations at the Birmingham Congress. Miss Lowe, superintendent of health visitors for the Warwickshire County Council, urged the necessity for a Paternity and Child Welfare Act, and strongly advocated the passage into law of the Bastardy Bill now before Parliament. Mrs. E. Eve spoke of the part of the father in maternity and child welfare being necessarily rather in the background, and made some suggestions for bringing it out more prominently. But some of them do not appear very promising. There are certain respects in which a father may be compelled—and is compelled by law—to observe responsibilities to his home; but there are a great many responsibilities which he ought to assume which no law could ever compel him to assume. The decent man will make a decent father, and in so far as he fails it may be attributed to ignorance. Nothing will definitely succeed in making an undesirable man a good father if his characteristics are not in that direction. That, of course, is a platitude of human nature. Mrs. Eve's plan for the better sort who approve of welfare work is to get them, if possible, to have short talks at their clubs on infant welfare! It is hard to ponder over this without a smile. Another plan is an occasional "At home" and exhibition of welfare work to which wives and husbands should be invited. This is not the sort of thing that the average husband will do. He regards it as outside his line, however deep his interest in his children. The first person who can interest a husband in welfare work—which in individual cases means proper care of one's own children—is the wife, and there are existing agencies for approaching the wife if she is not what she might be. If the husband must be approached, it can only be in his home, and, in any event, any husband who could be persuaded to attend exhibitions would be the very sort who is in the least need of it. But health workers and health visitors could do a good deal in interesting fathers in what is being done for their children, and that is a reasonable point which Mrs. Eve includes among her other suggestions. Dr. Bostock also emphasised this point.

It must not be overlooked in these discussions of the father's responsibilities to his family that he is bound fairly closely by law. Mr. Charles Ekin, Chairman of the Birmingham Branch of the N.S.P.C.C., observed that, quite apart from the moral responsibility, which most men observe by natural instinct, the Legislature has always, in dealing with the rights of legitimate children, made the father legally liable for their maintenance. A woman living with her husband may leave her home and incur no legal penalty, unless, perhaps, in the case of a suckling babe; but if the father leaves his family without making provision for them he would be liable to prosecution for neglect under the Children Act, or, if they became chargeable to the rates, to be sent to prison as a rogue and a vagabond. Further than this, if a woman neglected her home and family the law would also regard the father as responsible if he did not take measures for the protection of the children and obviate the consequences of the mother's acts. Even in cases where the parents are living apart under a deed of separation, or by an order of a court of summary jurisdiction, the court has held the father to be liable to punishment if he has failed to make the payments allotted for their maintenance, and it is a question whether he would not be responsible under the Act in the event of the children being generally neglected by the mother in cases where the father is living elsewhere and the mother has physical possession of the children. The tendency of the law is that children should not be deprived of the protection of the father, in spite of the fact that he is not living with his wife, although if the mother were to create a position which deprived him of any reasonable chance of doing his duty he could not be convicted of wilful neglect. The legal position of the father has always been one of greater responsibility.

